# Histidine Focused Covalent Inhibitors Targeting Acetylcholinesterase: A Computational Pipeline for Multisite Therapeutic Discovery in Alzheimer’s Disease

**DOI:** 10.1021/acschemneuro.5c00508

**Published:** 2025-09-25

**Authors:** Sadia Jaman, Salsabil Fatima Tasmi, Imrul Shahriar, Mohammad A. Halim

**Affiliations:** † Division of Computer-Aided Drug Design, The Red-Green Research Centre, BICCB, 16 Tejkunipara, Tejgaon, Dhaka 1215, Bangladesh; ‡ James Tarpo Jr. and Margaret Tarpo Department of Chemistry, 311308 Purdue University, West Lafayette, Indiana 47907, United States; § Department of Chemistry and Biochemistry, 271594 Kennesaw State University, Kennesaw, Georgia 30114, United States

**Keywords:** acetylcholinesterase (AChE), Alzheimer’s disease, epoxide warhead, multitargeted inhibitors, histidine, molecular dynamics

## Abstract

Alzheimer’s disease affects over 10% of individuals above the age of 65, yet current treatments offer only limited and temporary relief. Acetylcholinesterase, a key enzyme in neurotransmitter breakdown, also contributes to disease progression by promoting β-amyloid aggregation. While previous studies have focused on the catalytic serine, a key proton transfer residue, His447 remains unexplored as a potential covalent binding site. In this study, we aim to interrupt the activation of Ser203 by covalently modifying His447, thereby shutting down the entire catalytic process. Here, we reported a computational pipeline to identify epoxide-based small molecules that covalently engage His447 and modulate AChE activity. From a curated library of >7,000 epoxides, three ligands (L5, L6, L7) were selected via covalent docking, molecular dynamics simulations, and drug-likeness profiling. Microsecond-scale simulations revealed stable binding across multiple subsites, with L5 exhibiting the most consistent RMSD and compact *R*_g_ values. Covalent engagement of L5 and L6 induced modest shifts in His447 (2.48 and 1.43 Å), whereas L7 maintained apo-like geometry. Furthermore, ADMET predictions indicated favorable profiles, with no cardiotoxicity risk. Our findings highlight His447 as a novel covalent target in AChE and support further in vivo investigation of the specificity and inhibitory mechanisms of these ligands.

## Graphical abstract

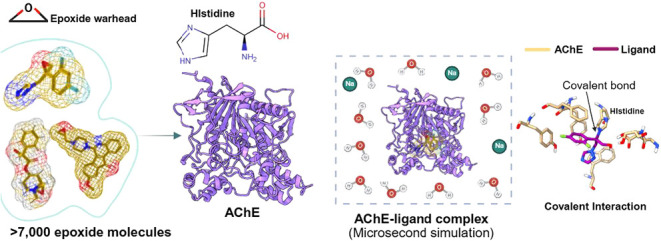

## Introduction

In 2022, the World Health Organization estimated that 55.2 million people worldwide were living with dementia. This number is projected to rise to 78 million by 2030, representing an increase of over 40% within just eight years.^1^ As the primary cause of dementia, AD accounts for 50–75% of all dementia cases.^2,3^ This slowly progressive neurodegenerative disorder affects approximately 10% of individuals aged 65 and above.^4^ Alzheimer’s disease is characterized by the formation of amyloid plaques due to unusual aggregations of β-amyloid proteins between nerve cells and the presence of neurofibrillary tangles resulting from the hyperphosphorylation of tau proteins.^5,6^ Another pathological hallmark of Alzheimer’s disease is the interaction between AChE and amyloid-β (Aβ), which promotes the aggregation of Aβ into insoluble plaques. Specifically, Aβ binds to the peripheral anionic site (PAS) of AChE through hydrophobic and aromatic interactions at the gorge entrance. The deep (∼20 Å) catalytic gorge is surrounded by a strong electrostatic field and large dipole moment, which acts as a funnel to attract charged molecules. Although Aβ exhibits a weaker dipolar character, its distinct positive and negative charge distributions are strongly attracted to AChE’s electrostatic field, promoting peptide stacking on the AChE surface. This interaction lowers the nucleation barrier, shortens the lag phase, and accelerates fibrillogenesis. The resulting plaques contribute to neuronal loss in the hippocampus, a region essential for memory, learning, and emotional regulation.^7–9^ Among older individuals with dementia, approximately 25% are prescribed antidementia medications, including acetylcholinesterase inhibitors (AChEIs).^10^

AChE, a cholinergic serine hydrolase enzyme found at postsynaptic neuromuscular junctions, is encoded by a gene on chromosome 7 at 7q22. The binding site of AChE includes several subsites: the catalytic triad (CAT), acyl pocket, oxyanion hole, anionic site, and peripheral anionic site (PAS).^11,12^ The catalytic triad of acetylcholinesterase (AChE) comprises Ser203, His447, and Glu334. Ser203 and His447 directly participate in the catalytic reaction, while Glu334 stabilizes the transition state through electrostatic interactions. Ser203, containing a hydroxyl group (−OH), acts as a nucleophile, attacking the target molecule, acetylcholine. His447, due to its nucleophilic properties, accepts a hydrogen atom from Ser203. This histidine residue facilitates proton transfer between the oxygen atoms of serine and acetylcholine, resulting in the cleavage of acetylcholine and the formation of an acylated serine intermediate. Subsequently, water molecules interact with His447, aiding in the deacetylation of acylated serine, which leads to the release of acetate and choline and returns the enzyme to its inactive state. Additionally, the anionic subsite is essential for stabilizing binding of acetylcholine to AChE. A three-pronged oxyanion hole helps to hold the acetyl group in position during these bond-making and bond-breaking processes. The acyl pocket of AChE determines the specificity of the acetyl esters. This pocket facilitates accommodation and interaction with acetyl groups, enabling the enzyme to selectively bind and hydrolyze acetyl esters. The peripheral anionic site (PAS) is linked to the acyl pocket via a deep hydrophobic gorge. The hydrophobic nature of this gorge guides and directs the binding of specific ligands to the PAS, thereby influencing the enzyme’s activity and regulation.^11,13,14^

Various acetylcholinesterase (AChE) inhibitors, including galantamine, donepezil, tacrine, and rivastigmine, are available for AD therapy. Donepezil, approved by the FDA, acts on both the anionic subsite and the PAS of AChE. Galantamine binds to the active-site gorge. Carbamate derivatives such as physostigmine, eptastigmine, and rivastigmine interact with both the esteric and anionic sites at the lower part of the gorge. Tacrine forms ionic bonds with specific amino acids of the anionic site.^15–17^ However, these drugs are effective in treating only mild to moderate AD, with limited efficacy and adverse side effects, including cholinergic issues, hepatotoxicity, and poor bioavailability. Though targeting the catalytic triad for drug development presents a promising avenue, very few drugs currently employ this approach.^18,19^ Recent drug discovery strategies, such as organophosphates, target Ser203 of the catalytic triad by covalently phosphorylating its hydroxyl group.^20^ However, at physiological pH, Ser203 alone is catalytically inert and depends on His447 for deprotonation.^21^ Despite this central role in the proton transfer mechanism, His447 has remained largely unexplored as a therapeutic target.

Therefore, in this study, we investigated epoxide covalent inhibitors against His447 of AChE. A Targeted Covalent Inhibitor (TCI) is characterized by its ability to bind covalently to a specific molecular target and suppress its biological function.^22^ They have specific functional groups that form stronger bonds with the target than noncovalent interactions. This increases their potency compared to noncovalent analogs and maintains prolonged inhibition of the target until protein degradation and regeneration.^23^ The nucleophilic nitrogen of the imidazole side chain of His447 can attack the electrophilic epoxide ring, forming a covalent bond that inhibits AChE activity by blocking the active site and prolonging acetylcholine signaling at synapses. To test this hypothesis, we employed advanced computational chemistry techniques to evaluate the binding affinity and inhibitory potential of these epoxide-based inhibitors. To the best of our knowledge, this is the first computational chemistry–driven exploration of His447 as a covalent target in AChE (see Figure 1). Notably, targeting histidine is an attractive approach due to its frequent proximity to protein-small molecule ligands and the nucleophilicity of its imidazole side chain.^24^

**Figure 1:**
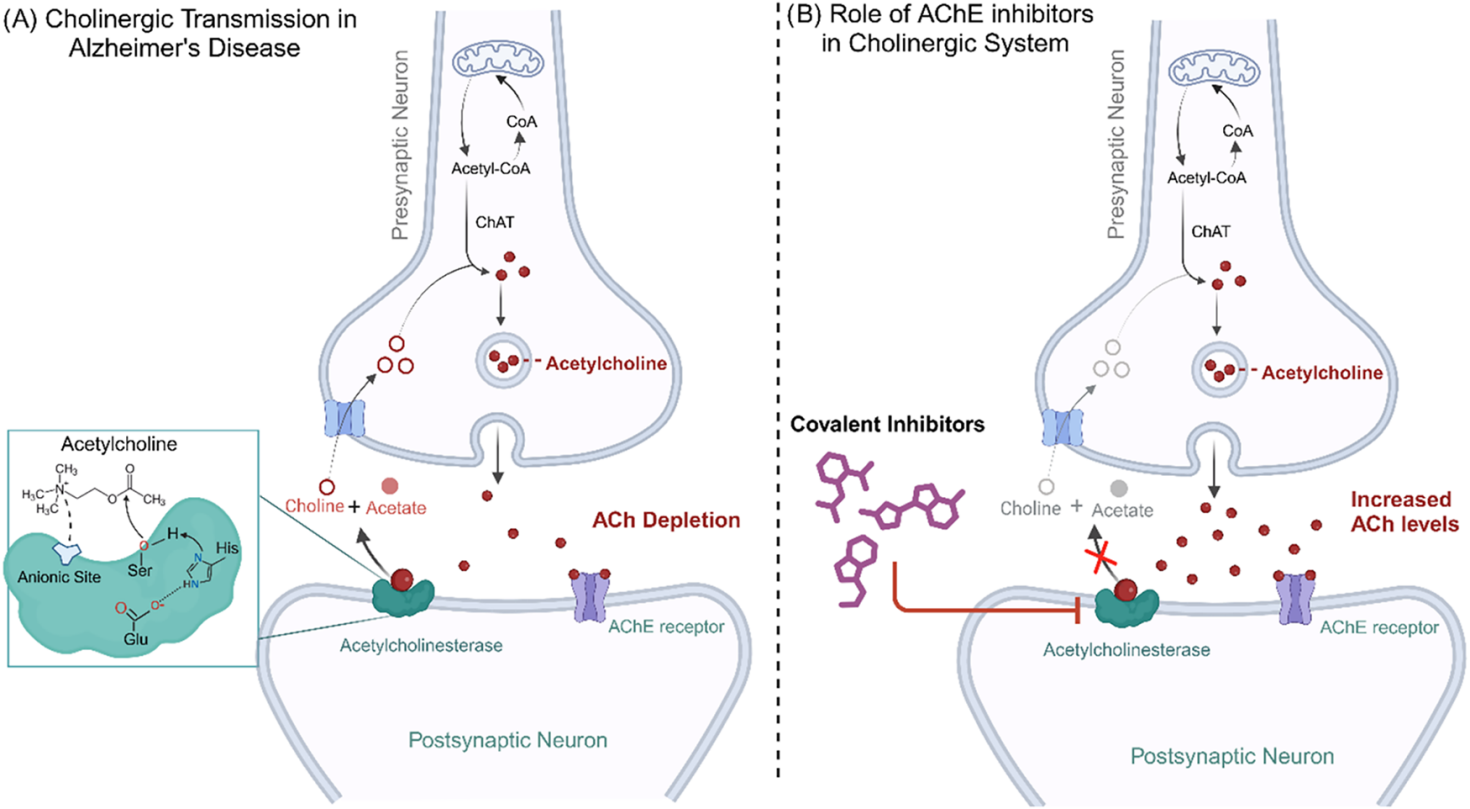
(A) Cholinergic synapse. When the presynaptic membrane undergoes depolarization, the synaptic vesicles release acetylcholine into the synaptic cleft through exocytosis, which binds to acetylcholine receptors. This process is terminated by AChE, which breaks down acetylcholine into choline and acetate, and choline is reabsorbed in the presynaptic neuron. (B) Effect of AChE inhibitors. Covalent inhibitors irreversibly bind to AChE with high affinity, blocking the acetylcholine interaction and preventing its hydrolysis.

## Results and Discussion

### Structure Analysis of AChE

Comparative sequence analysis, supported by insights from recent studies,^25–28^ identified key functional site residues of AChE (Figure S1). The anionic subsite consists of Trp86 and Phe338, while the oxyanion hole is formed by Gly121, Gly122, and Ala204. The acyl pocket is composed of Trp236, Phe295, and Phe297. Furthermore, the peripheral anionic site (PAS) includes Tyr72, Asp74, Tyr124, Glu285, Trp286, Trp337, and Tyr341, highlighting its potential role in substrate binding and allosteric modulation.

### Identification of High-Affinity AChE Ligands through Structure-Based Virtual Screening

In the past three decades, covalent drug design has gained increasing attention as it offers unique pharmacodynamic efficacy and enhanced potency.^29^ In our study, covalent docking-based virtual screening predicted 3,613 ligands that are capable of binding to His447. The top 12 were selected based on the highest docking scores and binding conformations (Table S1), and further analyzed in pose prediction mode. The binding affinities and protein interactions of these 12 ligands are summarized in Table S2. During the selection process, particular emphasis was placed on assessing whether these ligands could simultaneously engage additional functional sites, including PAS, oxyanion holes, and anionic sites, while maintaining covalent interactions with His447. Lead compounds were identified based on more negative binding affinities and stronger interactions with AChE.

Most of the compounds exhibited docking scores ranging from −9.5 to −8.67 kcal/mol. One exception, ChEMBL2408903, showed a lower binding score of −7.21 kcal/mol. All 12 ligands formed a covalent bond with His447. Among them, L5 exhibited the highest binding affinity (−10.1 kcal/mol) and formed a strong hydrogen bond with His447. Although L3, L9, and L12 demonstrated high binding affinities (−9.18, −9.20, and −9.03 kcal/mol, respectively), their overall interactions with the protein were weaker compared to those of other candidates. Notably, L2, L6, L7, and L8 exhibited strong binding interactions. Furthermore, L7 and L11 showed strong interactions with the peripheral anionic site residues through hydrogen bonding and hydrophobic interactions.

### Covalent Docking Studies

To refine the selection of candidate hits, covalent docking in pose prediction mode was carried out for the top 12 ligands. Among these, L5, L6, and L7 with covalent docking scores of −9.25, −8.87, and −9.02 kcal/mol, respectively, were selected as final candidates based on their docking affinities, MMGBSA scores, binding modes assessments, and ADMET calculation. MMGBSA calculations revealed significant binding free energies for L5 (−67.37 kcal/mol) and L6 (−62.95 kcal/mol), whereas L7 showed a comparatively lower value of −58.42 kcal/mol (Figure 2A).

**Figure 2:**
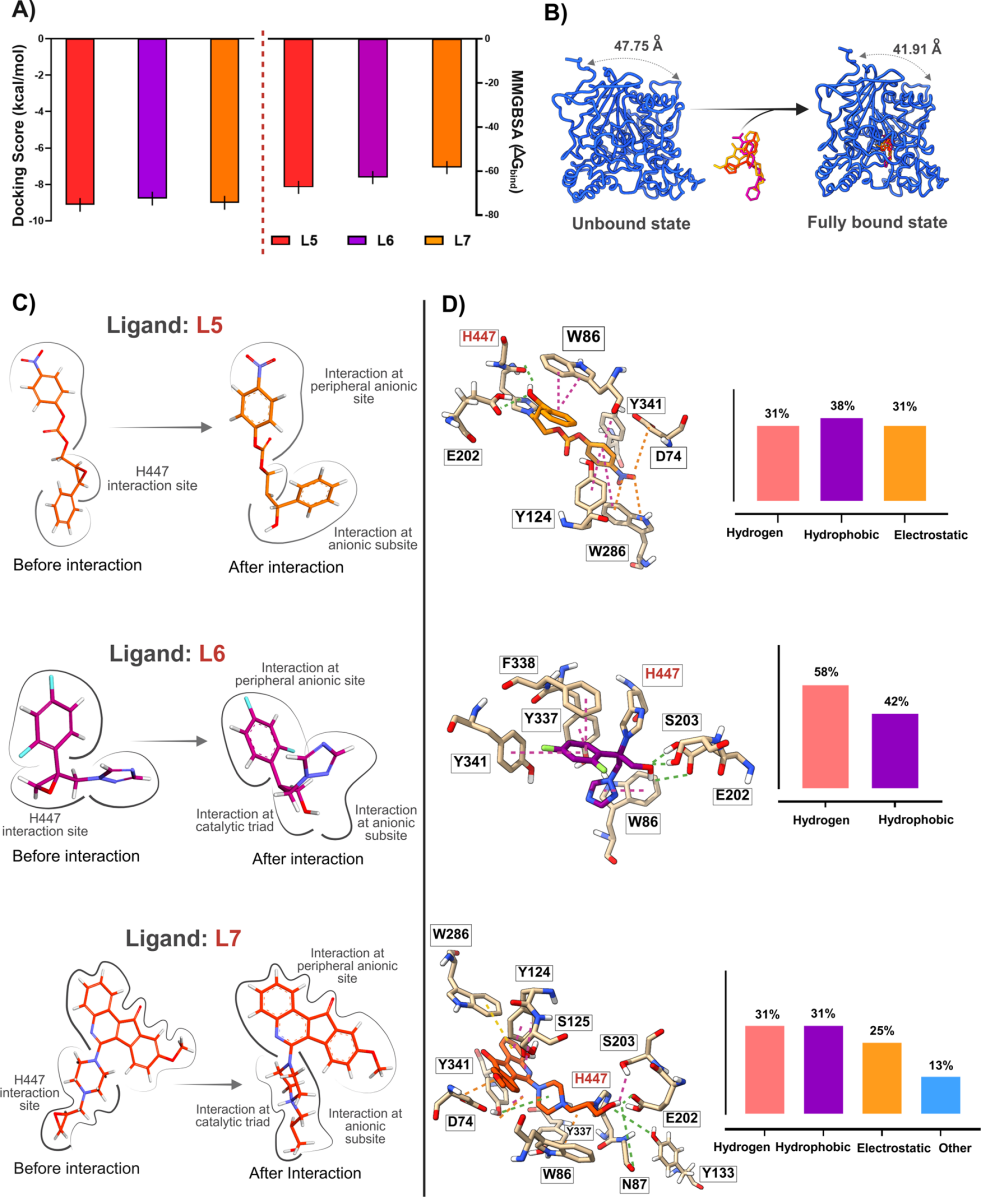
(A) Covalent docking affinity scores and binding free energy values of L5, L6, and L7 (left). Based on affinity score, the ligands were ranked in the order of L5 > L7 > L6, while binding free energies (Δ*G*) ranked the ligands as L5 > L6 > L7. (B) AChE-ligand interactions. Average distances between the Cα atoms of L9 and K496 are displayed. (C) Structural changes of ligands showing the predicted binding modes within the binding pocket. Interaction of nucleophilic H447 with the epoxide ring leads to subsequent ring opening. (D) Hydrogen bonds, hydrophobic interactions, and electrostatic interactions are shown in green, pink, and orange, respectively. A covalent bond between H447 and the ligands is clearly visible.

The binding of three ligands induced a global structural shift, reflecting an overall compaction or reorganization of AChE that may enhance ligand specificity by optimizing the geometry of key functional sites (Figure 2B). Consistent with the initial hypothesis, the nucleophilic nitrogen of His447 facilitated ring opening of the epoxide moiety in all three ligands, forming covalent adduct (Figure 2C). Additionally, the compounds interacted with key regions of AChE, including PAS, acyl pocket, and anionic subsite through a combination of hydrogen bonding, hydrophobic interactions, and electrostatic interactions (Figure 2D). For L5, the imidazole ring of His447 formed a C–H···π interaction with the ligand, maintaining a torsional angle of 113.66° and a distance of 2.52 Å. The aromatic rings of L5 also participated in π–π stacking interactions with Trp286 and Trp86, as well as hydrophobic interactions with the aromatic rings of Tyr124 and Tyr341. Moreover, the oxygen atom of the opened epoxide formed a conventional hydrogen bond with the hydroxyl group of Tyr124 at a distance of only 1.7 Å. The electrostatic interaction between the nitro group of L5 and the carboxylate side chain of Asp74 further stabilized the ligand at PAS.

On the other hand, the difluorobenzene moiety of L6 played a crucial role in mediating π–π stacking interactions with the aromatic side chains of PAS residues Tyr337 and Tyr341, as well as the anionic subsite residue Phe338. Additionally, the triazole group of L6 formed hydrophobic interactions with Trp86. Interestingly, the opened epoxide oxygen of both L6 and L7 formed a conventional hydrogen bond with Ser203, indicating a strong and favorable alignment within the catalytic pocket. Similar to L5, the quinoline scaffold of L7 strongly interacted with PAS. However, L7 maintained a slightly greater distance from this site, with an average distance of ∼4.4 Å. Furthermore, the pyrazine ring of L7 formed an electrostatic interaction with Trp86.

### Drug-Likeness and Pharmacokinetics Evaluation

To evaluate the drug-likeness of the 12 designed compounds, their physicochemical properties were assessed against established filters, including Lipinski’s rule of five, Ghose, Veber, Egan, and Muegge criteria. Among these, L5, L6, and L7 demonstrated optimal ADMET profiles, which are discussed in detail herein. All three compounds exhibited favorable oral bioavailability, characterized by molecular weights (MW) < 500 Da, topological polar surface areas (TPSA) < 130 Å^2^, hydrogen bond donors (HBD) < 5, hydrogen bond acceptors (HBA) < 10, and the number of rotatable bonds (nRB) < 9. Notably, these compounds showed no PAINS and were fully compliant with all five drug-likeness filters (Tables S3 and S4). According to ADMET predictions (Table S5), all three compounds exhibited favorable absorption properties, including human intestinal absorption >30% and Caco-2 permeability >0.90. Notably, L6 and L7 displayed blood–brain barrier (BBB) permeability, suggesting potential central nervous system penetration.

All the lead compounds were presumed to have no significant interaction with P-glycoprotein, a membrane transporter that prevents drug accumulation in the brain.^30^ However, L7 was identified as a potential substrate for P-gp, which might influence its plasma concentration and biological efficacy. In addition, none of the compounds inhibited members of the CYP family, except for CYP3A4 in L5 and L7, which might have certain effects. All the ligands were nonsensitizing to skin and did not inhibit CYP2D6, indicating minimal risk of dermatological reactions and drug–drug interactions mediated by CYP2D6, respectively. Notably, L5 did not exhibit hepatotoxic effects, indicating the safety concerning liver function. None of the compounds inhibited hERG I, thereby mitigating concerns regarding potential cardiac side effects. The low clearance rate of L5 suggested that it might be eliminated from the body more slowly, leading to prolonged drug exposure. L6 exhibited moderate clearance, while L7 demonstrated high clearance, indicating a shorter duration of action. However, in silico ADMET relies on statistical models, QSAR, or machine learning algorithms trained on existing data sets. These models are prone to overfitting and often fail to generalize to novel chemotypes, leading to false positives and false negatives. Thus, experimental validation through in vitro assays, such as Caco-2 permeability, microsomal stability, and hERG inhibition, together with in vivo pharmacokinetic profiling, is essential to confirm these predictions.^31^

### Molecular Dynamics, Structural Stability, and Flexibility Assessment of Covalently Bound L5, L6, and L7 to AChE

To investigate the structural stability and interaction patterns of L5, L6, and L7, we performed 1 μs molecular dynamics simulations of the complexes. The RMSD profiles of AChE were found to be in the range of 0.5–3.5 Å (Figure 3A), indicating overall structural stability of the complexes. As reported in previous studies, lower RMSD fluctuations indicate favorable conformational stability.^32^ Among the evaluated compounds, L5 exhibited superior stability, maintaining an average RMSD of 2.8 Å throughout the simulation. However, a slight increase was observed after 900 ns, reaching a peak at approximately 3 Å. In contrast, both L6 and L7 showed low RMSD values during the first 270 ns, followed by a gradual increase thereafter. Between 610 and 700 ns, the RMSD of L6 reached a maximum of 3.2 Å and subsequently stabilized after 730 ns. While for L7, greater fluctuations were recorded between 730 and 850 ns, followed by a further increase beyond 900 ns, suggesting reduced structural stability of AChE with this ligand.

**Figure 3:**
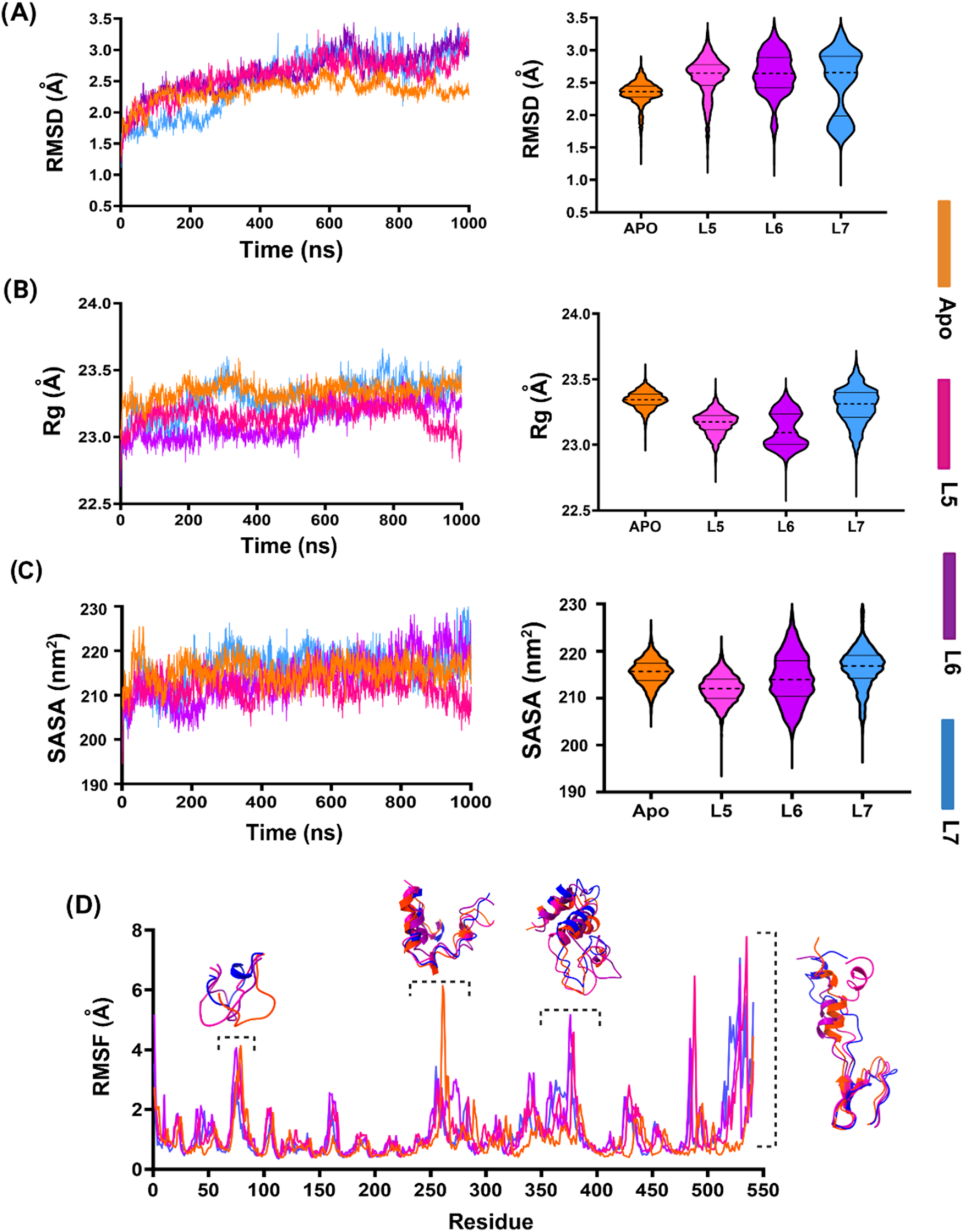
Molecular dynamics simulation of three ligand-AChE complexes. (A) RMSD of α carbon of AChE, (B) *R*_g_ of the complexes, and (C) SASA. The scatter plots illustrate the structural deviations over time, while the violin plots display the distribution and probability density of the data. The dashed lines represent the medians, and the black lines indicate the quartile ranges. (D) Structural superimpositions above the RMSF peak showed regions of higher flexibility induced by ligand binding compared with Apo AChE.

The radius of gyration (*R*_g_) analysis was evaluated to assess the compactness and rigidity of the complexes. Lower *R*_g_ indicates more compactness and stable protein structure.^33^ As shown in Figure 3B, the AChE and L5-AChE complexes revealed almost similar types of unimodal violin plots, which suggested that the binding of L5 did not induce significant conformational changes in AChE, and this protein remained stable and compact in both free and complexed states. In fact, the *R*_g_ decreased from 23.1 to 22.9 Å after 880 ns. For L6-AChE complex, the average *R*_g_ value remained stable at ∼23.2 Å during the initial 520 ns but increased over the simulation time. For L7-AChE complex, the *R*_g_ value was lower than apo-AChE for the first 220 ns, then merged with the apo between 275 and 520 ns. From 730 to 820 ns, the *R*_g_ increased higher than the apo form, reflecting a potential loss of structural compactness. Overall, *R*_g_ analysis showed the average order of compactness and rigidness: L5-complex (23.3 Å) > L6-complex (23.35 Å) > apoprotein (23.45 Å) > L7-complex (23.58 Å).

The solvent-accessible surface area (SASA) was calculated to determine how the surface of the compound is exposed to solvent (Figure 3C). A decrease in SASA upon ligand binding often suggests that the ligand is partially or fully buried within the protein.^34^ In this study, the average SASA value for L5-AChE and L6-AChE complexes had slightly lower SASA values (mean 212 and 214 nm^2^, respectively) compared to the apo form (mean 217 nm^2^). This reduction indicates that both L5 and L6 could be well-set within the binding pocket of AChE. Specifically, the decrease in the L5-AChE complex is likely attributed to extensive hydrophobic interactions with key residues such as Trp, Phe, Tyr, and Leu. In the case of L6, approximately 75% of the interactions involved hydrophobic residues, leading to reduced solvent exposure of these residues. In contrast, the L7-AChE complex had a higher SASA value (218 nm^2^), potentially because of the rigid and bulky structure of the L7. This structural feature might have limited occupancy within the active site, thereby enhancing the solvent-exposed surface area, as also reported by Konstantinidis et al.^35^

To assess how ligand binding influences protein structural flexibility at the single-amino acid level, residue-specific root-mean-square fluctuation (RMSF) was analyzed (Figure 3D). Higher RMSF values indicate higher flexibility in the protein–ligand complex. Overall, notable fluctuations were observed in structural regions: 71–86, 254–270, and near the C-terminus (511–541) across all complexes. The catalytic triad residues His447, Ser203, and Glu334 showed low RMSF values for all three ligands, remaining within 0.66 Å. Specifically, RMSF values at His447 were 0.64 Å for L5, 0.66 Å for L6, and 0.608 Å for L7, indicating strong ligand interactions. The L5-AChE complex exhibited low residue fluctuations (<2.5 Å), except for the C-terminal residues, showing its maximum of 6.5 Å. However, in comparison with other complexes, L5 demonstrated higher fluctuations in Phe297 (acyl-binding pocket) and in Glu285 and Trp286 (PAS), while showing lower fluctuations in Ala121 and Ala122 (oxyanion hole). L6-AChE exhibited higher fluctuations in Ala121 and Ala122 relative to the other complexes, indicating greater flexibility in this region. On the other hand, the L7-AChE complex had slightly higher fluctuations in Asp72, Asp74, Tyr124, Trp337, and Tyr341 (PAS). Surprisingly, all ligand-bound AChE complexes exhibited lower RMSF values between residues 350 and 400 compared to the apo form, indicating more rigidity and formidability in this region.

### Molecular Mechanism of AChE Inhibition by L5, L6, and L7

During the MD simulation, multiple conformational snapshots were generated to capture key transition states and intermolecular interactions, providing insights into critical binding events in Figure 4. Throughout the simulation, the (2*S*,3*S*)-epoxide ring of L5 formed a covalent bond with the imidazole ring of His447. Additionally, the carbonate moiety of L5 had a tendency to form hydrogen bonds with His447, suggesting a secondary stabilizing interaction. In the early stages, L5 predominantly interacted with PAS residues through hydrophobic contacts, and as the simulation progressed, these interactions were further enhanced at the anionic subsite by 1000 ns.

**Figure 4:**
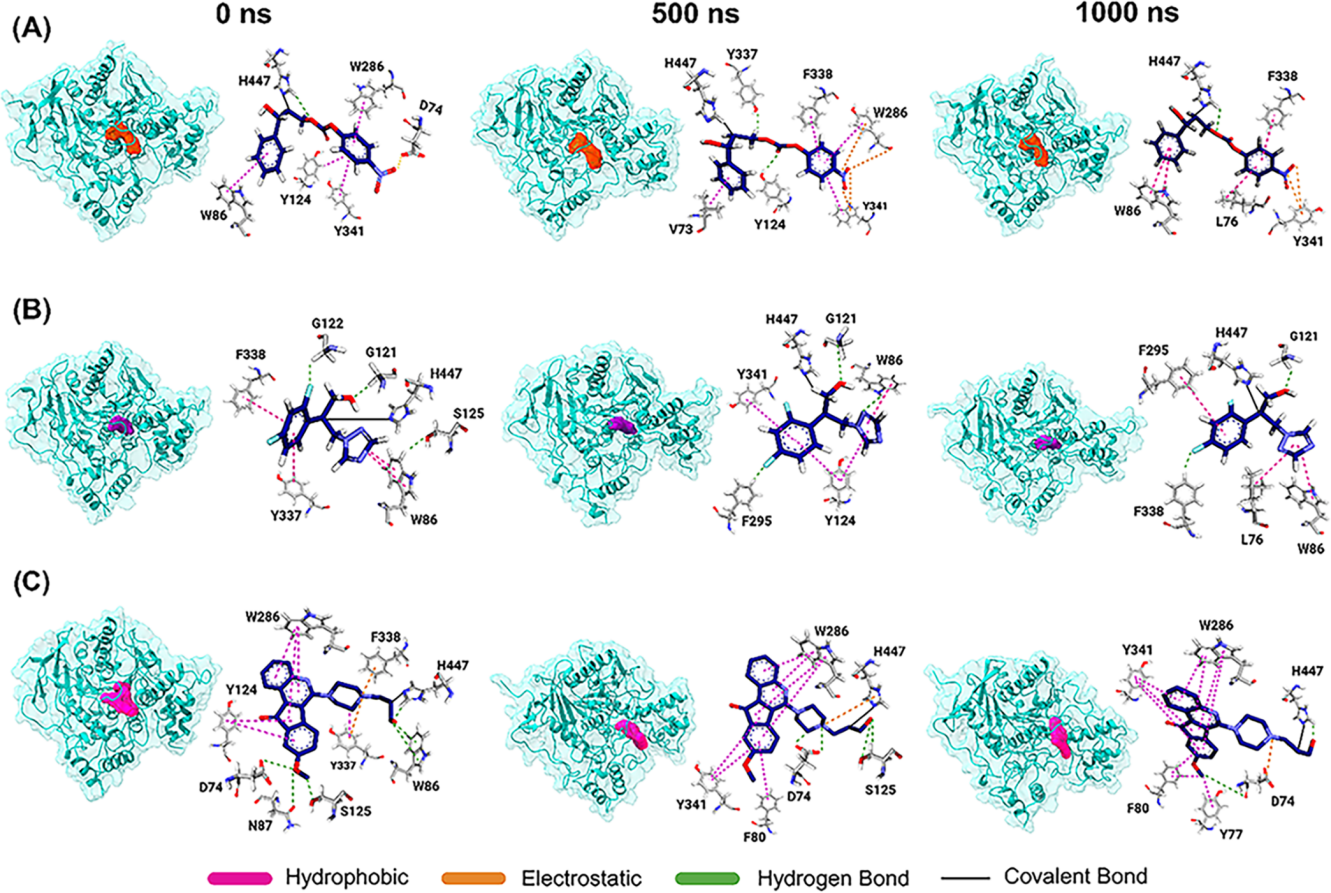
Representative snapshots of the four complexes. Snapshots of the simulation were taken in 200 ns intervals starting from 0 to 1000 ns of simulation. (A) AChE-L5 (orange); (B) AChE-L6 (purple); (C) AChE-L7 (deep pink).

Notably, the PAS of AChE plays a crucial role in amyloid-β (Aβ) fibrillogenesis, facilitating the formation of AChE–Aβ complexes that accelerate Aβ peptide assembly and contribute to the deposition of mature senile plaques in the AD brain. Given the role of the PAS in this pathological process, it serves as a primary target for FDA-approved drugs such as donepezil (IC_50_ = 5.7 nM).^36,37^ However, our findings suggested that L5 exhibited dual functionality, not only inhibiting ACh hydrolysis but also interfering with Aβ aggregation and offering a potential therapeutic advantage over single-site inhibitors.

While covalently bound to His447, the opened epoxide oxygen of L6 formed a hydrogen bond with the oxyanion hole residue, Gly121, located near the catalytic triad. It is worth mentioning that the carbonyl oxygen of ACh has been reported to interact with the oxyanion hole residues, as previously reported.^26^ Further investigations are needed to determine whether L6 competes with ACh for binding to the AChE. In addition, the difluorobenzene ring of L6 played a critical role in interactions with the acyl pocket residue Phe295 and the PAS residues, while its triazole ring engaged with the anionic subsite residue Trp86, which indicates a multitarget binding profile.

L7, an indeno­[1,2-*c*] quinoline derivative, demonstrated strong electrostatic interactions between its quinoline scaffold and the key PAS residues Trp286 and Tyr341. At 500 ns, its heterocyclic moiety formed electrostatic interactions with covalently bound His447. Furthermore, L7 maintained stable hydrogen bonds with Trp86 throughout the simulation. Indeno­[1,2-*c*]­quinoline derivatives are well documented for various biological activities, including antiproliferative, anti-inflammatory, antiviral, and antimycobacterial properties; however, their potential application in neurological disorders remains underexplored, representing a promising avenue for future research.^38,39^

### Free Energy Landscape (FEL) and Principal Component Analysis (PCA) Interpretation

The free energy landscape (FEL) shows thermodynamic stability and kinetic pathways by mapping energy minima, transition barriers, and conformational states.^40^ The shape and depth of the energy basins are visualized using a color gradient ranging from red to blue, where deep blue regions correspond to low-energy, well-localized clusters—indicative of more folded, energetically favorable conformations and a high degree of structural stability. In contrast, red-shaded regions represent higher-energy, less folded conformations that are typically associated with transient or unstable structural states.^41,42^ The FEL of the apo form displayed a long, narrow, low-energy basin, implying that the unbound protein adopts a well-defined conformation or a set of closely related structural states (Figure 5). On the other hand, the L5-AChE complex exhibited a slightly rough landscape, with small and discrete low-energy spots, which indicated that L5 binding induced only minor structural rearrangements, maintaining a near-native protein conformation, as supported by the *R*_g_ analysis. In the case of L6, it revealed additional metastable states, but the very less transition barrier <1.0 kcal/mol between the ensemble states suggested a stable conformation of the complex, confined within an energy basin and transitioning smoothly between closely related substates. In comparison, the L7–AChE complex displayed a broader distribution of high-energy regions (red) compared to both the apo form and other ligand-bound complexes, reflecting greater conformational heterogeneity and the presence of multiple transient, less stable structural states, suggesting more flexible and dynamic behavior upon L7 binding.

**Figure 5:**
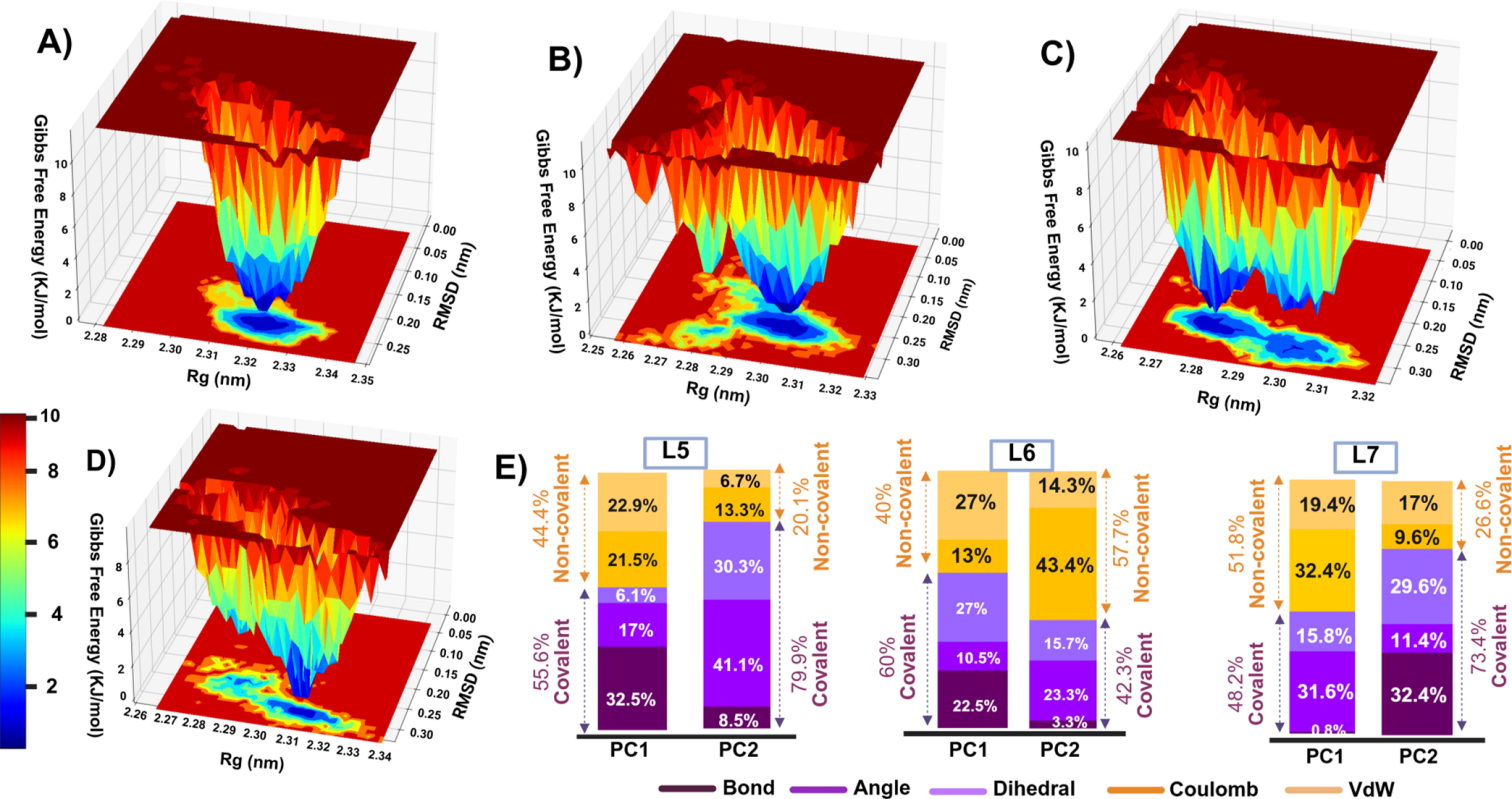
FEL and PCA plot analyses for AChE complexes. Three-dimensional contour map representing FEL of (A) Apo AChE, (B) L5-AChE complex, (C) L6-AChE complex, and (D) L7-AChE complex plotted against RMSD and *R*_g_. (E) PCA stacked bar plot illustrating variance interpretation of L5-AChE, L6-AChE, and L7-AChE complexes. In the L5 and L7 complexes, covalent terms contributed more than 70% in PC2, showing that the covalent linkage strongly restricted bond and torsional flexibility.

Principal component analysis (PCA) was conducted to identify key structural and energetic variations among ligand-bound AChE complexes (Figure 5E). In the decomposition of force-field energies for PCA bonded terms, including bond stretching, angle bending, and dihedral (torsional) rotations, represent as covalent contributions. In contrast, nonbonded terms, such as van der Waals (vdW) and electrostatic (Coulomb) interactions, arise from interactions between atoms that are not directly covalently linked and are hence considered noncovalent contributions.^43^ For the L5–AChE complex, covalent interactions dominated the variance, contributing 55.6% to PC1 and 79.9% to PC2, indicating that bond, angle, and dihedral motions strongly governed the conformational dynamics of the AChE upon L5 binding. In the L6–AChE complex, covalent terms accounted for 60.0% of PC1 variance, driven by dihedral contributions (27.0%), indicating initial structural adaptation via torsional flexibility. However, PC2 exhibited a shift, with noncovalent terms, particularly van der Waals and Coulombic interactions, suggesting that electrostatic stabilization and hydrophobic contacts enhanced binding pocket affinity as the L6 progressed. For the L7–AChE complex, covalent dominance persisted, with 48.0% in PC1 and 73.4% in PC2. The substantial dihedral contribution in both PCs reflected the restricted torsional flexibility. This restriction reduced conformational fluctuations in the active-site gorge and stabilized the geometry of the catalytic triad. Overall, covalent attachment not only anchored the ligands but also transmitted rigidity through the AChE backbone, locking the enzyme into a less flexible, inactive conformation.

### Superimposition of FEL-Derived Stable Conformations to Identify Major Structural Changes

Compared with the reference apo AChE structure, the ligand-bound complexes largely retained their overall secondary structure, as evidenced by structural overlap among the models (Figure S4). However, notable changes were detected in the C-terminal region, where the α-helix was partially lost and replaced by a coil-like conformation, as confirmed by RMSF analysis. Additionally, we also observed structural rearrangements in the key functional regions (Figure 6). For instance, a significant conformational shift occurred in the 73–89 loop, which includes key residues of the PAS and the catalytic anionic subsite, such as Tyr72, Asp74, and Glu86. In the apo form, this loop is oriented inward-facing, whereas the binding of L5, L6, and L7 induced an outward displacement of up to ∼8.12, ∼10.54, and ∼13.81 Å, respectively. Interestingly, in the segment spanning residues 334–343, the backbone of the apo state exhibited a more open and twisted conformation, which underwent a transition to a more compact, intercalated structure upon ligand binding. The PAS residues Tyr337 and Tyr338 were directly involved in this transition. This conformational tightening at the entrance of the catalytic gorge may reduce flexibility and enhance ligand affinity, consistent with the behavior of known PAS-targeting inhibitors. Attention was also given to His447 containing loop (residues 434–450). In the apo state, this loop adopted a compact conformation. Ligand binding induced outward shifts, with maximum Cα displacements of ∼4.42 Å (L5) and ∼5.36 Å (L6), while the L7-bound complex retained a conformation closely resembling the apo form. His447 itself shifted by 2.48 Å in the L5-bound and 1.43 Å in the L6-bound structures. Interestingly, in the L7-bound complex, the loop segment spanning residues 285–289, including the PAS residues Glu285 and Trp286, underwent a distinct transition. While this region in the apo state formed an extended loop with side chains-oriented outward, it adopted a twisted, β-turn-like conformation in the ligand-bound state. This rearrangement likely contributes to local stabilization and reduced conformational entropy, suggesting an inhibitor-induced ordering effect potentially linked to the irreversible covalent binding mechanism.

**Figure 6:**
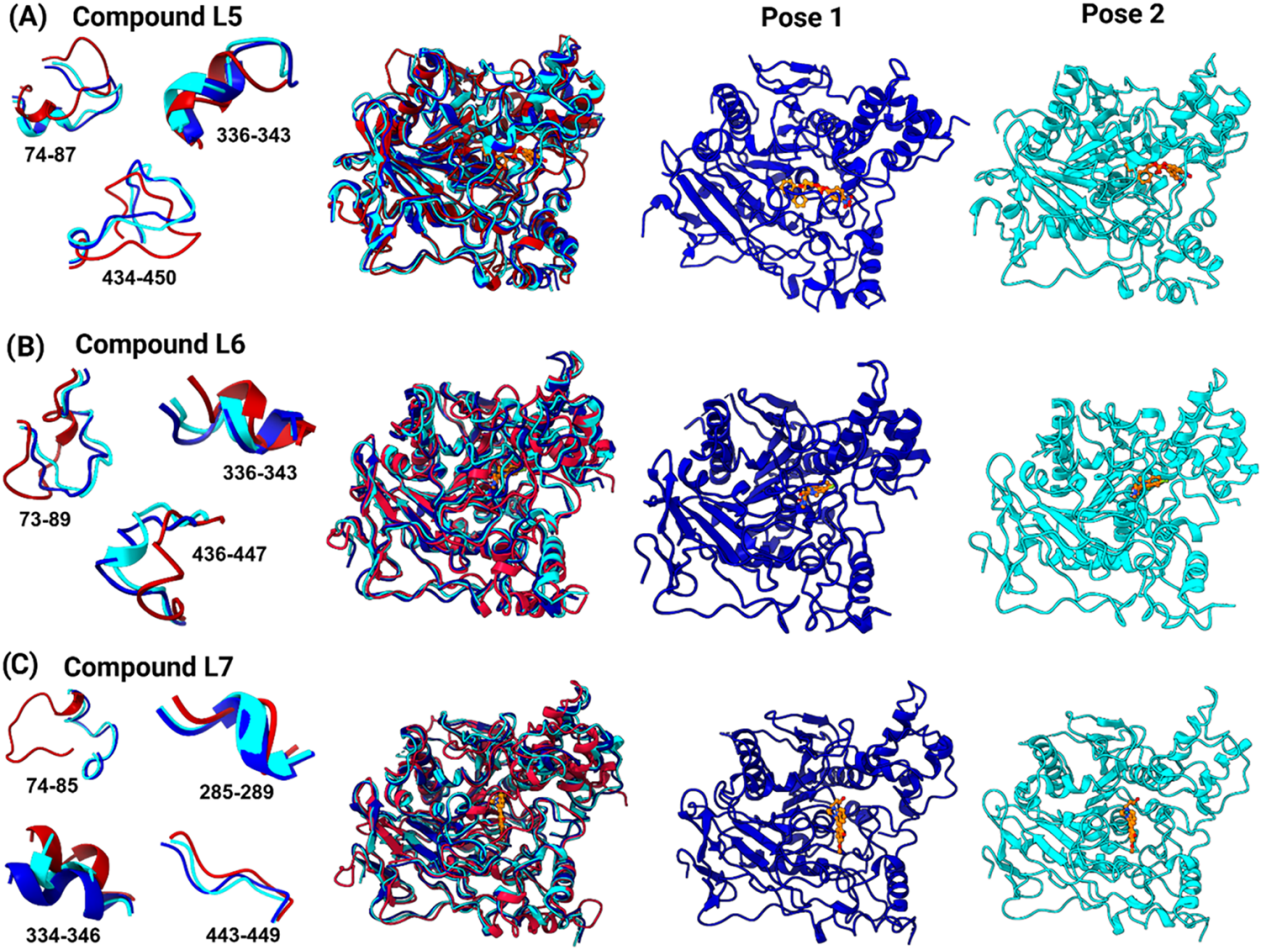
Superimposed representations of the two most energetically favorable structures of (A) the L5–AChE complex, (B) the L6–AChE complex, and (C) the L7–AChE complex, aligned with the apo AChE structure. The apo form is colored red, while the ligand-bound conformations are colored blue and cyan.

## Future Aspects

While our computational findings are promising, we acknowledge that these techniques cannot fully capture transition state energetics, competing reaction pathways, or the influence of solvent and local microenvironments. Therefore, experimental validation remains essential to confirm the covalent bond formation. To address these limitations, covalent labeling–mass spectrometry (CL-MS) or LC-MS/MS could provide residue-level binding site information. To evaluate solvent and microenvironment effects, covalent adduct formation can be monitored by LC–MS/MS under varying pH and ionic strength, while enzyme inhibition kinetics can be measured under identical conditions. Site-directed mutagenesis is also a powerful approach, as site specificity is assessed by mutating the nucleophile, which should abolish adduct formation and potency.^44^ Structural methods, such as X-ray crystallography and NMR spectroscopy, allow direct visualization of the covalent adduct at the binding site as well as chemical shift changes consistent with covalent bond formation. In addition, Activity-Based Protein Profiling (ABPP) employing covalent activity-based probes (ABPs) represents a powerful chemo-proteomic strategy to characterize covalent modifications. Such approaches are increasingly important for identifying covalently modified off-target proteins at an early stage, thereby reducing risk and accelerating the development of covalent therapeutics.^44^

## Conclusions

Current efforts in drug development increasingly emphasize covalent inhibitors due to their high potency, evidenced by low IC_50_ values, and sustained target engagement, which together allow for lower dosing and reduced administration frequency relative to noncovalent agents. This study employed a comprehensive computational pipeline to screen a large library of epoxide warheads targeting the catalytic residue His447 of AChE. Key observations include: (1) L5, a nipecotic acid derivative, interrupts acetylcholinesterase activity and amyloid-β aggregation, (2) L6 interacts with the catalytic triad, acyl pocket, PAS, oxyanion hole, and anionic subsite, demonstrating multisite inhibitory potential, (3) L7, featuring a quinoline core, interacts with PAS residues and covalently binds His447, suggesting potential for neurological applications; and (4) all three lead compounds exhibit effective oral bioavailability, with L6 and L7 showing the ability to permeate the blood–brain barrier. Furthermore, the results revealed a notable PAS-like conformational change in the 334–343 loop of AChE, which reduces flexibility at the catalytic gorge entrance and enhances ligand affinity. We believe that our study will encourage researchers to explore neurotoxicity studies, behavioral assessments in disease models, and real-time imaging of the distribution of these compounds in the brain.

## Methods

### Preparation of Protein and Ligands

The three-dimensional crystal structure of human AChE (PDB ID: 4EY7, resolution: 2.35 Å) was obtained from the Protein Data Bank.^19^ Using Swiss-PDB Viewer (version 4.1.0), we optimized the structure to its lowest energy state and added any missing hydrogen atoms.^45^ PyMol (version 2.6.0) was employed to remove all hetero atoms, water molecules, and inhibitors from the structure.^46^ Notably, reported residues of the anionic subsite, oxyanion hole, acyl pocket, and PAS of AChE vary across the studies. To maintain the accuracy, we performed sequence alignment using well-characterized torpedo species (PDB ID: 1EVE, resolution: 2.5 Å) through ESPript 3.0 and selected the key residues based on alignment results and recent literature studies. It is worth mentioning that the structure of human AChE is nearly identical to torpedo AChE.^28,47^

A chemical library was created consisting of 7104 epoxide-containing compounds (Supplementary File 2). These compounds, including FDA-approved drugs, ligands, pesticides, and phytochemicals, were collected from PubChem, BindingDB, ChEMBL, DrugBank, MedChemExpress, ZINC, and ChemSpider. The ligands were checked, and any hetero atoms and nonspecific molecules were removed by BIOVIA Discovery Studio Visualizer.

### Virtual Screening and Covalent Docking

Covalent docking-based virtual screening (CovDock-VS) was performed to screen 7104 compounds against AChE using the Covalent Docking tool in Maestro (fast mode). 51.5% (3613) of ligands demonstrating covalent bond formation were selected for further analysis. The top 12 candidates, based on docking scores and interactions, were subjected to additional covalent docking in pose prediction mode. For this process, His447 in the catalytic triad of AChE was selected as the reactive residue and the epoxide group in the ligands was chosen to react through an epoxide opening reaction. The Protein Preparation Wizard in Maestro was employed for the preparation and energy minimization of the crystal structure, while ligands were processed using Schrodinger’s LigPrep tool. Finally, the best 3 compounds were selected based on their affinity score (cdock affinity), MM/GBSA score, docking interactions, and ADMET study.

### ADMET and Drug-Likeness Properties

Evaluating ADMET parameters for each drug is crucial before entering preclinical trials to minimize the risk of withdrawal at various stages of preclinical and clinical development. Most of the pharmaceuticals heavily rely on early in silico prediction tools for this purpose.^48^ This study utilized SwissADME^49^ and pkCSM^50^ to investigate the drug-likeness and ADMET properties of the leading 12 compounds. These tools help researchers identify novel drug candidates, reduce the number of empirical experiments, and increase the success rate.^51^ Lipinski’s rule of five (ROF), Veber filter, Ghose filter, Egan filter, and Muegge filter were used as the primary screening criteria for drug-likeness. Secondary screening involved calculating ADMET properties, including bioavailability, brain penetration, oral absorption, carcinogenicity, and other characteristics of human intestinal absorption characteristics.

### Molecular Dynamics (MD), Free Energy Landscape (FEL), and Principal Component Analysis (PCA)

The top three ligands were subjected to 1 μs molecular dynamics simulations using the Desmond molecular dynamics package (commercial version, 2021), executed on a Linux-based workstation with NVIDIA CUDA-enabled GPU acceleration.^52^ System setup was performed by using the System Builder utility integrated within Desmond. The OPLS-2005 force field was employed as it is known for its ability to provide reliable parameters for proteins and ligands.^53^ The system was solvated using TIP3P water molecules within orthorhombic periodic boundary conditions for 10 Å buffer region to ensure that the system behaves as if it were part of an infinite crystal lattice, thus avoiding edge effects that could distort the simulation.^54^ To neutralize the system, Na^+^ and Cl^–^ ions were added to achieve a salt concentration of 0.15 mol/L and a pH of 7.4, thus, maintaining physiological conditions. Energy was minimized for 5 ns to remove any steric clashes or unfavorable interactions, providing a stable starting point for the MD simulation. An NPT ensemble with a Nose-Hoover thermostat and barostat was applied to keep the temperature at 310 K and pressure at 1 bar.^55^ Trajectory analysis was utilized to calculate the root-mean-square deviation (RMSD), root-mean-square fluctuation (RMSF), radius of gyration (*R*_g_), and solvent-accessible surface area (SASA). PyMOL, along with the Geo-Measures plugin, was employed to analyze FEL, and the secondary structure of the protein.

By applying the principal component analysis (PCA) method, different multivariate energy factors such as bond distances, bond angles, dihedral angle, planarity, van der Waals energies and electrostatic energies were calculated to understand the change of structural quality of protein in the presence of ligand during MD simulation.^56,57^ The last 100 ns of the MD trajectory data for apo (AChE) and four selected protein–ligand complexes were considered for PCA analysis. Bond length, bond angle, and dihedral terms were grouped as covalent contributions, whereas Coulombic and van der Waals terms were treated as noncovalent contributions. The covariance matrix of these descriptors was constructed and subjected to multivariate analysis in OriginPro. The squared loadings of each descriptor were calculated from the eigenvector coefficients, normalized within each PC, and expressed as percentage contributions. The relative contributions of covalent and noncovalent terms were then compared to assess their role in the essential dynamics of ligand–AChE complexes.

## Supplementary Material





## Notes

Additional computational and structural data supporting the study; 2D structures of the top 12 ligands, binding poses, binding affinities, and molecular interactions, as well as their physicochemical, medicinal chemistry, and ADMET properties; sequence alignment and functional site analysis of AChE orthologs, multiple docking pose predictions for the best three ligand–protein complexes, representative snapshots of these complexes, and analyses of secondary structure changes in the ligand-bound AChE (PDF)
Chemical library consisting of 7104 epoxide-containing compounds (SDF)

## Abbreviations

AChE: acetylcholinesterase
ACh: acetylcholine
ADMET: absorption, distribution, metabolism, excretion, and toxicity
BBB: blood–brain barrier
PAS: peripheral anionic site
