# Supplementary material for: Histidine Focused Covalent Inhibitors Targeting Acetylcholinesterase: A Computational Pipeline for Multisite Therapeutic Discovery in Alzheimer’s Disease
Source: ACS Chem Neurosci. 2025 Sep 25;16(20):4025–36. doi: 10.1021/acschemneuro.5c00508 (PMC12532204; doi:10.1021/acschemneuro.5c00508)
Supplement: Supplementary file 1 [file cn5c00508_si_001.pdf]

SUPPORTING INFORMATION

for

Histidine Focused Covalent Inhibitors Targeting Acetylcholinesterase: A Computational Pipeline for Multi-Site Therapeutic Discovery in Alzheimer's Disease

Sadia Jaman[1], Salsabil Fatima Tasmi[1], Imrul Shahriar[2], Mohammad A. Halim[3*]

[1]Division of Computer-Aided Drug Design, The Red-Green Research Centre, BICCB, 16 Tejkunipara, Tejgaon, Dhaka, 1215, Bangladesh

[2]James Tarpo Jr. and Margaret Tarpo Department of Chemistry, Purdue University, West Lafayette, IN 47907

[3]Department of Chemistry and Biochemistry, Kennesaw State University, Kennesaw, Georgia, 30114, USA

*Correspondence to: Mohammad A. Halim (E-mail: mhalim1@kennesaw.edu ORCID: https://orcid.org/0000-0002-1698-7044)

Table S1. 2D Structure of the best 12 ligands with binding pose and binding affinity

Ligand	Ligand id	2D Structure<br>Epoxide warhead 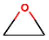	Binding Pose	Binding Affinity (kcal/mol)
L1	CHEMBL4077030	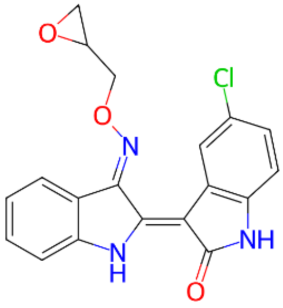	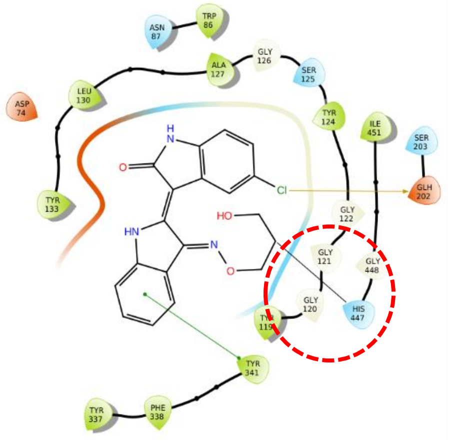	-8.73
L2	CHEMBL2408911	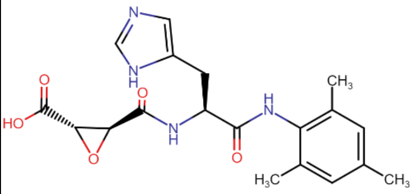	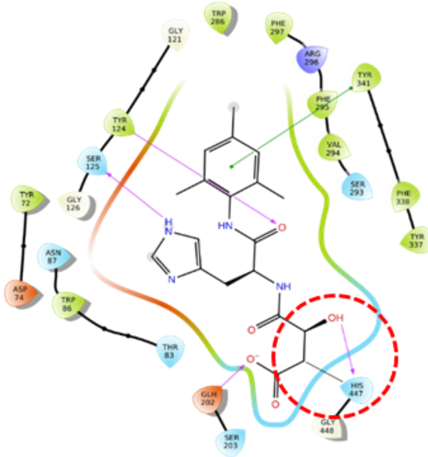	-8.05
L3	PubChem CID 123948005	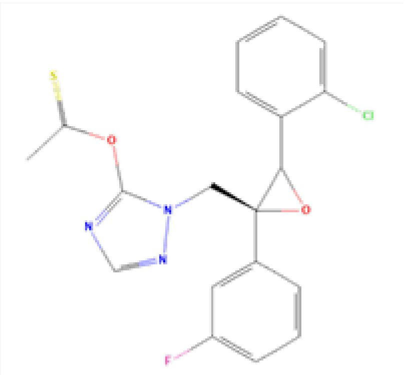	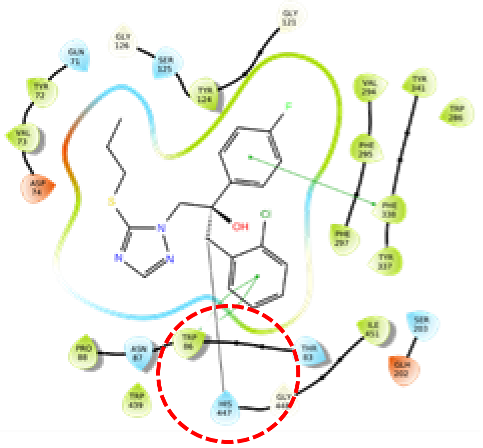	-9.18

Table S1. Continued

Ligand	Ligand id	2D Structure<br>Epoxide warhead 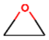	Binding Pose	Binding Affinity (kcal/mol)
L4	PubChem CID 90368786	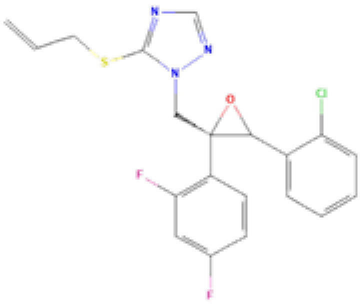	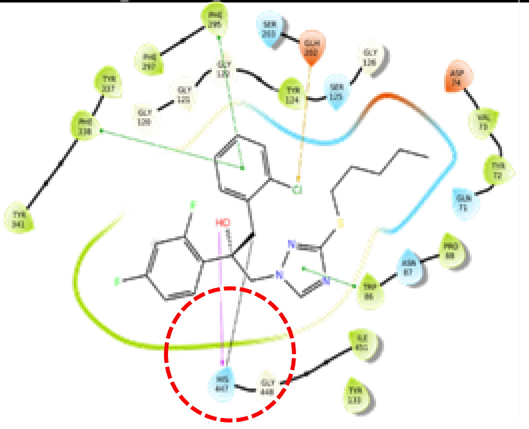	-8.25
L5	PubChem CID 16211069	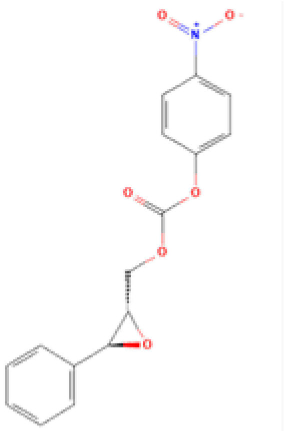	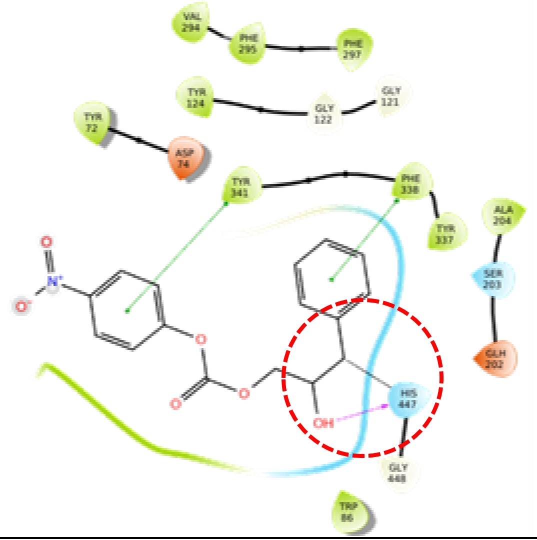	-10.1
L6	CHEMBL2062536	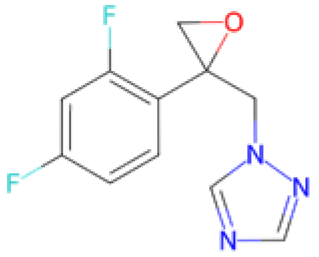	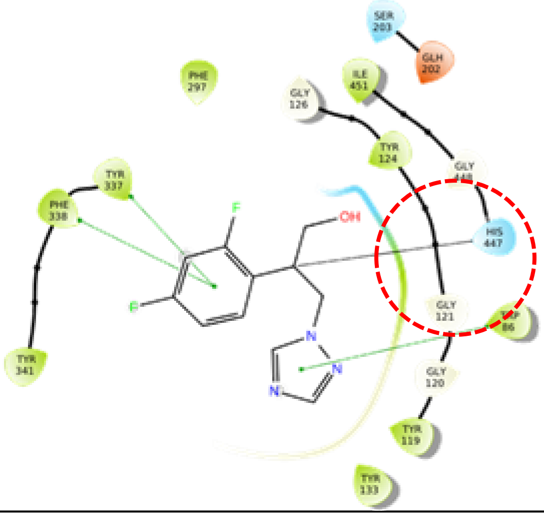	-8.67

Table S1. Continued

Ligand	Ligand id	2D Structure<br>Epoxide warhead 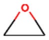	Binding Pose	Binding Affinity (kcal/mol)
L7	CHEMBL3585503	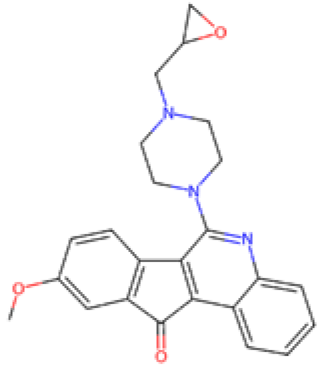	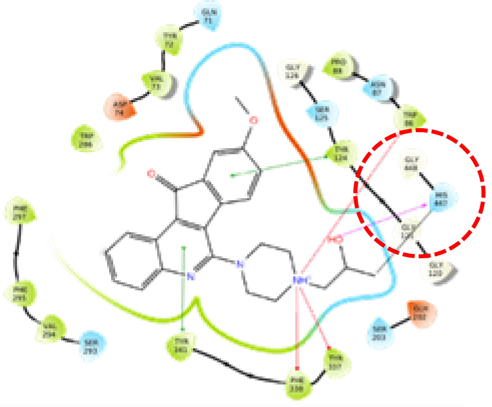	-8.17
L8	CHEMBL2408906	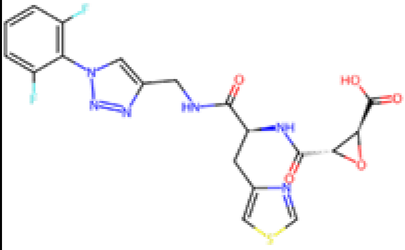	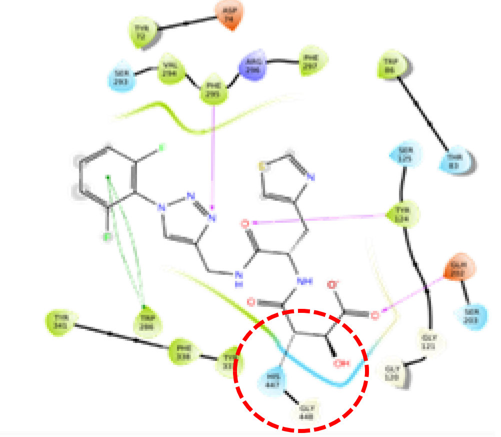	-9.505
L9	CHEMBL4090217	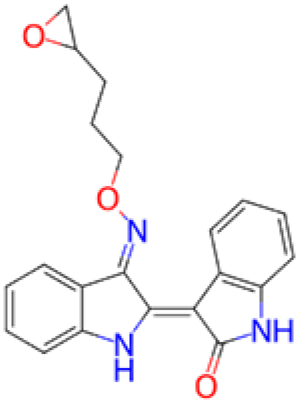	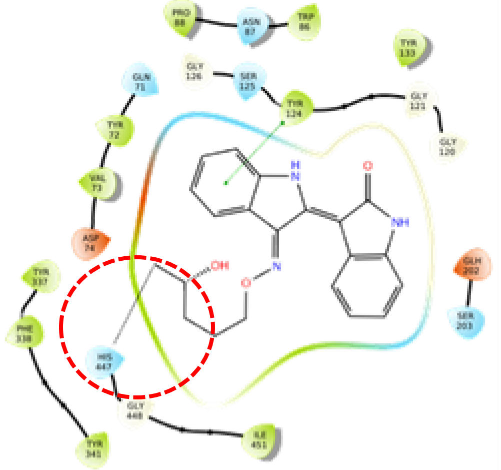	-9.20

Table S1. Continued

Ligand	Ligand id	2D Structure<br>Epoxide warhead 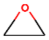	Binding Pose	Binding Affinity (kcal/mol)
L10	CHEBI:3388	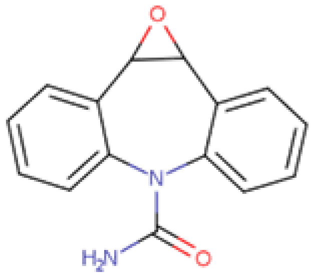	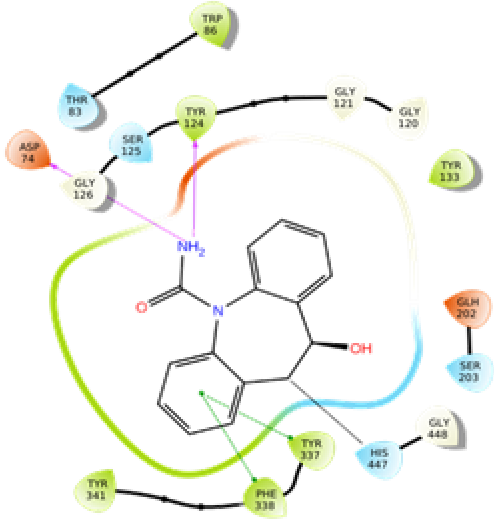	-8.13
L11	CHEMBL2408903	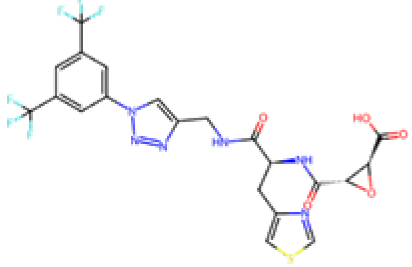	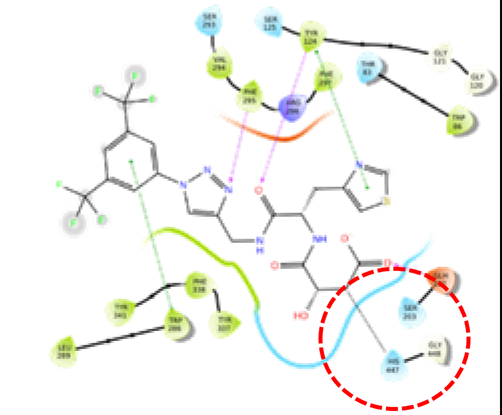	-7.21
L12	PubChem CID 3000322	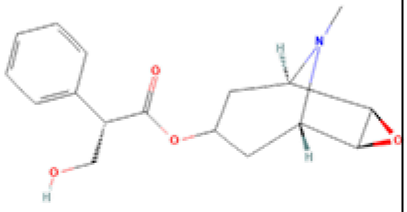	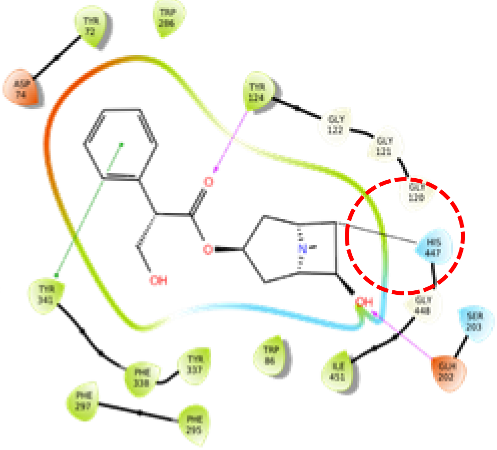	-9.03

Table S2. Interactions of the selected 12 ligands with the AChE.

Ligand Id	Interacting Residue	Bond Category	Bond Type
**L1**	TYR 341	Hydrophobic	Pi-Pi stacking
	GLH 202	Halogen	Halogen (Fluorine)
	HIS 447	Covalent	Unspecified residue
**L2**	SER 125	H	C
	TYR 124	H	C
	TYR 341	Hydrophobic	Pi-Pi stacking
	GLH 202	H	C
	HIS 447	H/Covalent	C/Unspecified residue
**L3**	TRP 86	Hydrophobic	Pi-Pi stacking
	TYR 341	Hydrophobic	Pi-Pi stacking
	GLY 121	Halogen	Halogen (Fluorine)
	HIS 447	Covalent	Unspecified residue
**L4**	TRP 86	Hydrophobic	Pi-Pi stacking
	TYR 337	Hydrophobic	Pi-Pi stacking
	PHE 338	Hydrophobic	Pi-Pi stacking
	HIS 447	Covalent	Unspecified residue
**L5**	TYR 341	Hydrophobic	Pi-Pi stacking
	PHE 338	Hydrophobic	Pi-Pi stacking
	HIS 447	H/ Covalent	C/Unspecified residue
**L6**	PHE 338	Hydrophobic	Pi-Pi stacking
	TYR 337	Hydrophobic	Pi-Pi stacking
	TRP 86	Hydrophobic	Pi-Pi stacking
	HIS 447	Covalent	Unspecified residue
**L7**	TYR 341	Hydrophobic	Pi-Pi stacking
	TYR 124	Hydrophobic	Pi-Pi stacking
	TRP 86	Other	Pi-cation
	TYR 337	Other	Pi-cation
	PHE 338	Other	Pi-cation
	HIS 447	H/Covalent	C/Unspecified residue
**L8**	TRP 286	Hydrophobic	Pi-Pi stacking
	TYR 124	H	C
	GLH 202	H	C
	PHE 295	H	C
	HIS 447	Covalent	Unspecified residue
**L9**	TYR 124	Hydrophobic	Pi-Pi stacking
	HIS 447	Covalent	Unspecified residue
**L10**	TYR 337	Hydrophobic	Pi-Pi stacking
	PHE 338	Hydrophobic	Pi-Pi stacking
	TYR 124	H	C
	ASP 74	H	C
	HIS 447	Covalent	Unspecified residue
**L11**	TYR 124	H/Hydrophobic	C/Pi-Pi stacking
	TRP 286	Hydrophobic	Pi-Pi stacking
	PHE 295	H	C

	GLH 202	H	C
	HIS 447	Covalent	Unspecified residue
**L12**	TYR 341	Hydrophobic	Pi-Pi stacking
	TYR 124	H	C
	GLH 202	H	C
	HIS 447	Covalent	Unspecified residue

Table S3. Physicochemical Properties

Lipinski's Rule	L5	L6	L7
**MW < 500**	315.28	237.21	401.46
**nRB < 9**	7	3	4
**HBA < 10**	6	5	5
**HBD < 5**	0	0	0
**TPSA < 130**	93.88 Å²	43.24 Å²	58.20 Å²

Table S4. Medicinal Chemistry

	L5	L6	L7
**Ghose Filter**	Yes	Yes	Yes
**Veber Filter**	Yes	Yes	Yes
**Egan Filter**	Yes	Yes	Yes
**Muegge Filter**	Yes	Yes	Yes
**PAINS alert**	0 alert	0 alert	0 alert

Table S5. ADMET Study of the Ligands

ADMET Study	Parameters	L5	L6	L7
**Absorption**	GI absorption	91.21%	98.606%	99.357%
	Caco2 permeability	1.146	1.273	1.094
	Skin Permeation	No	No	No
	P-gp Substrate	No	No	Yes
**Distribution**	BBB permeability	No	Yes	Yes
	CNS permeability	No	No	No
**Metabolism**	CYP3A4 substrate	Yes	No	Yes
	CYP2D6 inhibitor	No	No	No
**Excretion**	Total Clearance	0.133	0.262	1.053
**Toxicity**	Oral Rat Acute Toxicity (LD50)	2.426	2.17	2.315
	Hepatotoxicity	No	Yes	Yes
	hERG I inhibitor	No	No	No
	hERG II inhibitor	No	No	Yes
	Max. Tolerated Dose (human)	0.61	0.384	-0.064
	Skin sensation	No	No	No

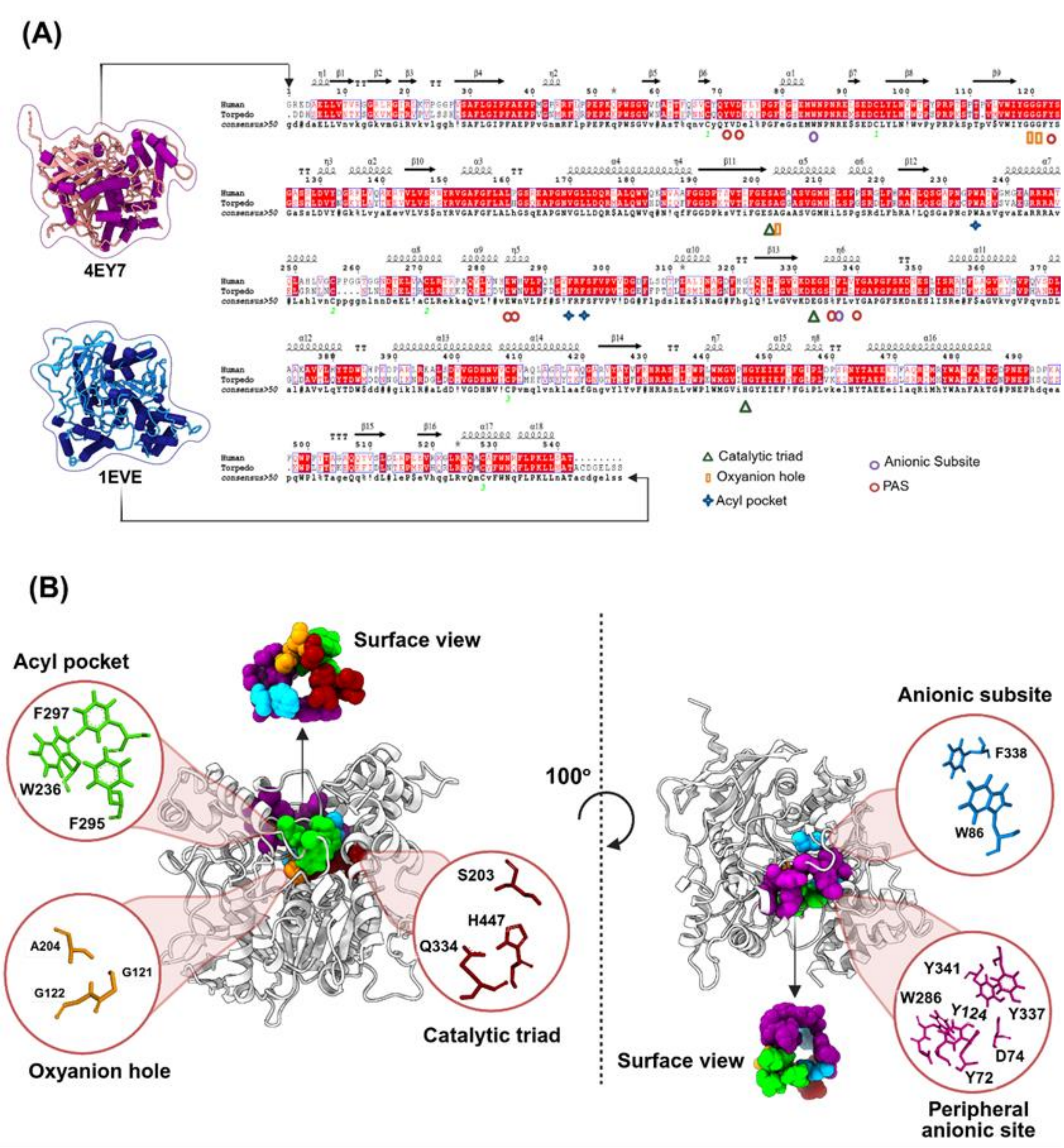

Figure S1. (A) Sequence alignment of AChE orthologs. Amino acid residues highlighted in blue open boxes represent similar residues, while those in red filled boxes denote identical residues. Conserved catalytic residues are marked with asterisks (*), whereas cation-binding residues are indicated by filled circles (●). Structural features are represented as follows: long squiggles for α-helices, arrows for β-strands, η for 3-10 helices, and 'TT' for strict β-turns. Sequence gaps are denoted by dots (.). (B) Analyzing functional sites of AChE. These sites play roles in ligand binding, acetyl group positioning, substrate interaction, and selective hydrolysis, ensuring efficient catalysis of ACh.

Ligand: L5

A) Docking Score: -5.423

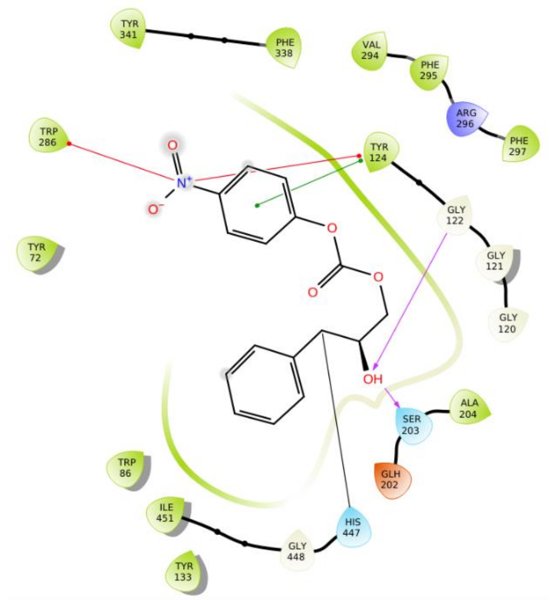

MMGBSA dG Bind: -62.48 kcal/mol

B) Docking Score: -5.84

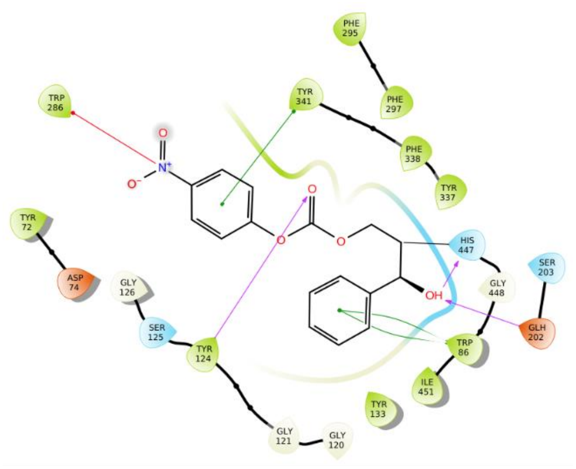

MMGBSA dG Bind: -59.1 kcal/mol

Ligand: L6

A) Docking Score: -5.586

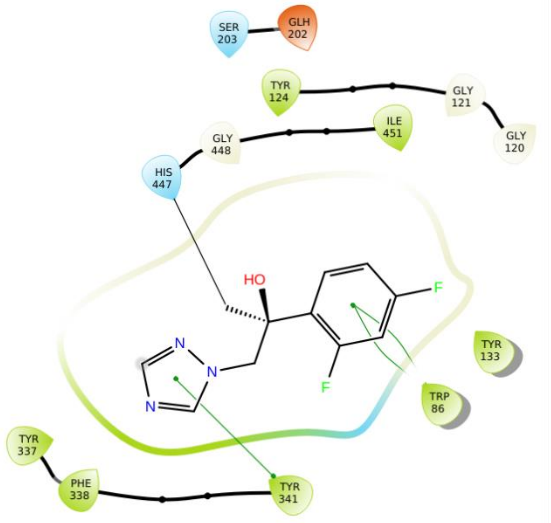

MMGBSA dG Bind: -52.89

B) Docking Score: -6.875

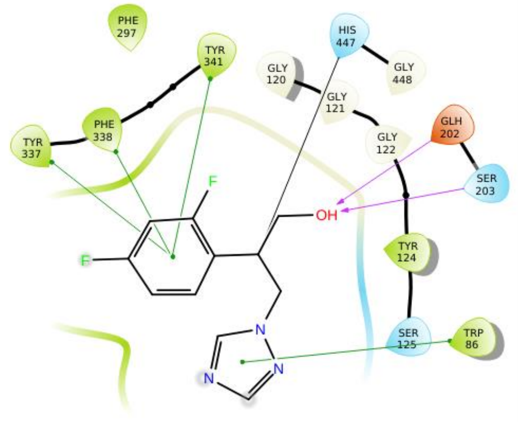

MMGBSA dG Bind: -59.95

Ligand: L7

A) Docking Score: -8.388

B) Docking Score: -9.714

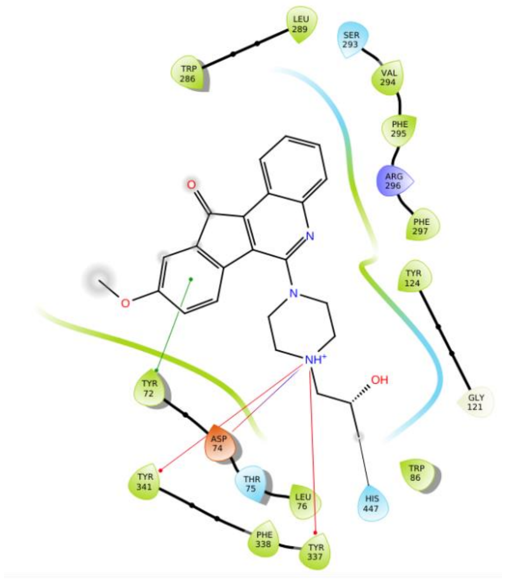

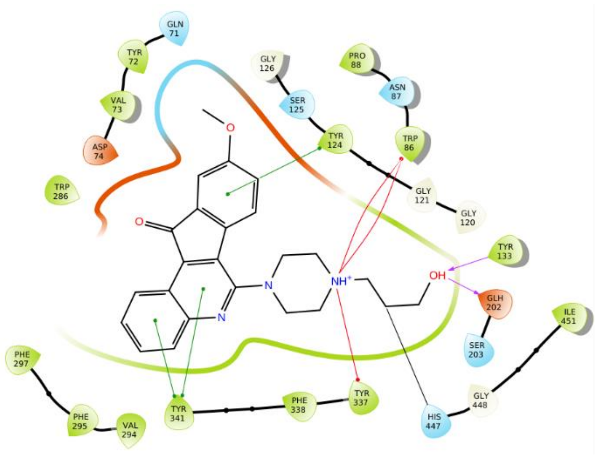

MMGBSA dG Bind: -55.73	MMGBSA dG Bind: -43.19

Figure S2. Different pose predictions of three ligand–protein complexes generated through covalent docking-based virtual screening. Each pose represents the best-ranked conformations of (A) AChE–L5, (B) AChE–L6, and (C) AChE–L7 based on docking scores and MM-GBSA ΔG bind values. Ligands were selected based on their covalent interaction with the catalytic triad, particularly His447.

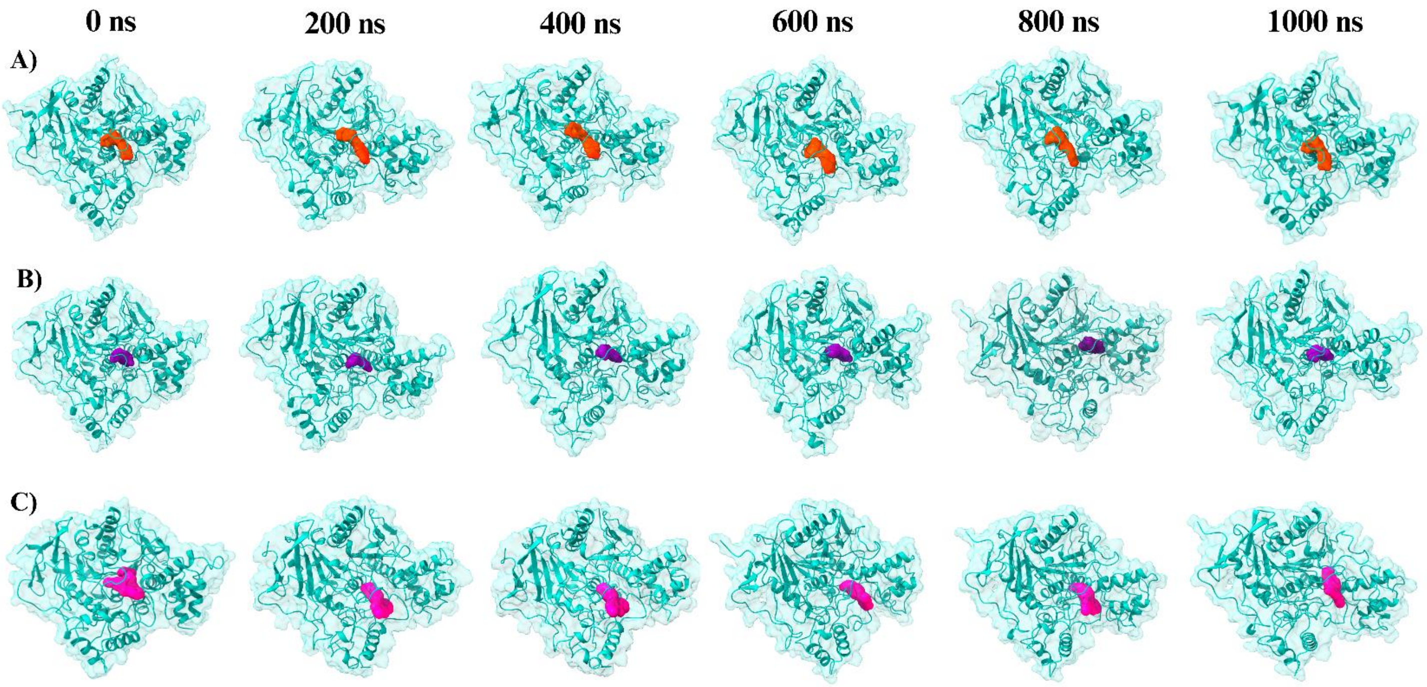

Figure S3. Representative snapshots of three complexes. Snapshots of the simulation were taken in 200 ns intervals starting from 0 to 1000 ns of simulation. (A) AChE-L5 (Orange); (B) AChE-L6 (Purple); (C) AChE-L7 (Deep Pink). Snapshots were taken using ChimeraX.

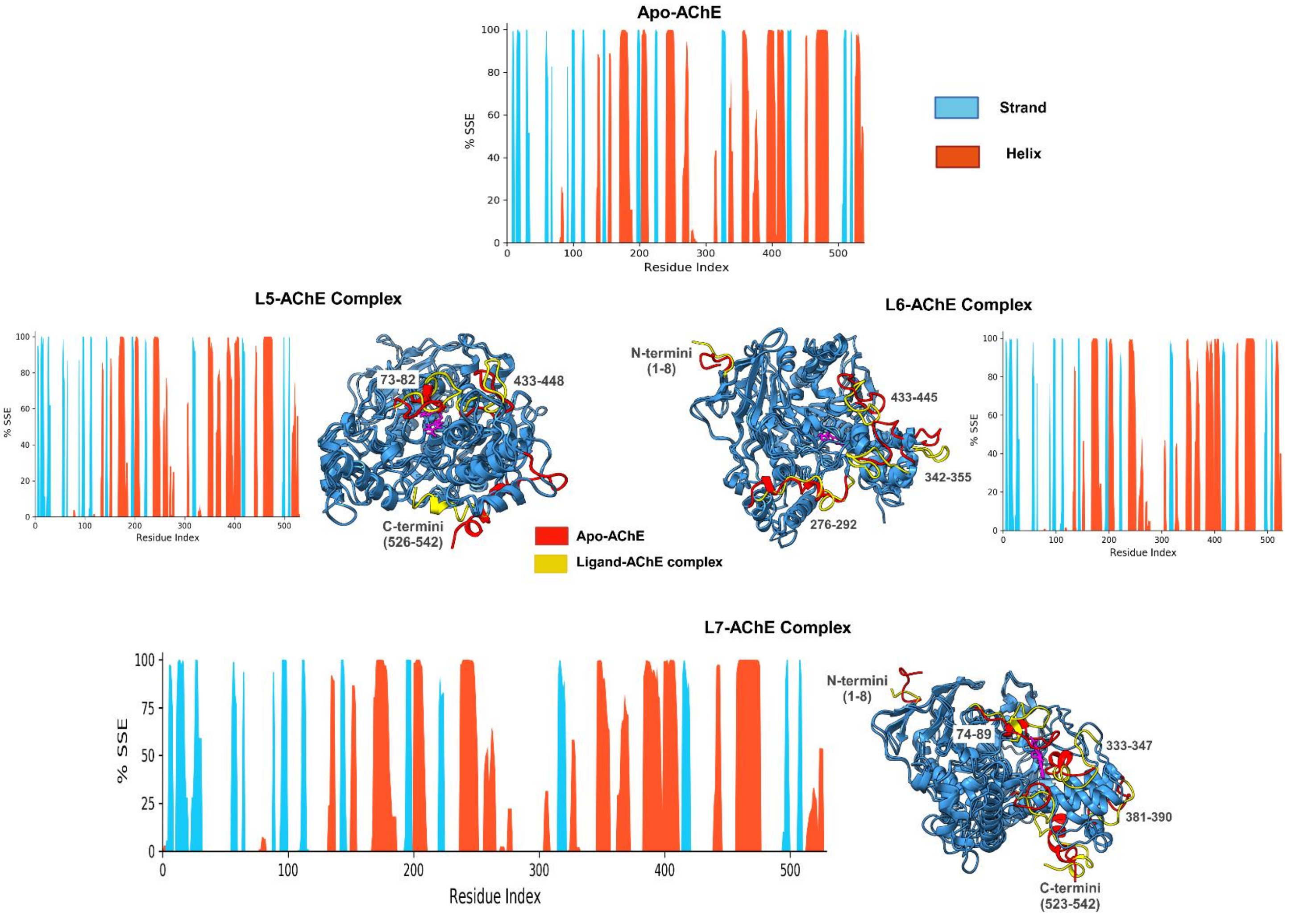

Figure S4. Ligand binding to AChE induces notable changes in its secondary structure, with the greatest changes observed for L7, followed by L6 and L5.
